# Growth and Yield of Purple Kculli Corn Plants under Different Fertilization Schemes

**DOI:** 10.3390/jof8050433

**Published:** 2022-04-22

**Authors:** Teresa Romero-Cortes, Lis Tamayo-Rivera, Mario A. Morales-Ovando, José E. Aparicio Burgos, Victor H. Pérez España, Martin Peralta-Gil, Jaime A. Cuervo-Parra

**Affiliations:** 1 Escuela Superior de Apan, Universidad Autónoma del Estado de Hidalgo, Carretera Apan-Calpulalpan, Chimalpa Tlalayote, Apan 43900, Hidalgo, Mexico; romero@uaeh.edu.mx (T.R.-C.); tamayo@uaeh.edu.mx (L.T.-R.); jose_aparicio@uaeh.edu.mx (J.E.A.B.); victorhugo_perez@uaeh.edu.mx (V.H.P.E.); martin_peralta10391@uaeh.edu.mx (M.P.-G.); 2 Sede Acapetahua, Universidad de Ciencias y Artes de Chiapas, Calle Central Norte s/n Entre 4ª y 5ª Norte, Acapetahua 30580, Chiapas, Mexico; mario.morales@unicach.mx

**Keywords:** *Zea mays*, bioinoculants, purple corn, *Trichoderma harzianum*

## Abstract

Globally, corn is the most economically important crop, surpassing other cereals of economic importance. However, the tillage methods, monoculture and the abuse of synthetic agrochemicals used in Mexico have led to the loss of fertility and soil yield. In this sense, the application of alternative fertilization methods based on chemical fertilizer, organic matter and biofertilizer, applied alone or in combination, can stimulate the defense systems of corn plants and increase their yield. Therefore, in this research, some fertilization schemes were tested on purple corn plants of the Kculli race through the evaluation of some growth and yield variables, as well as the subsequent evaluation of the chemical characteristics of the corn grain produced in each fertilization scheme. The results indicate highly significant differences (*p* ≤ 0.05) between treatments, for the different growth and yield variables studied. Of all the fertilization schemes evaluated, treatment T7 obtained the best grain yield of 6.19 ± 0.07 t ha^−1^, with respect to treatment T1 of 1.02 ± 0.01 t ha^−1^, as well as the highest protein content and starch quality. Being clear the positive effect of the adequate contribution of the macro and micronutrients used exerts on the corn crop in each of the fertilization schemes studied. On the other hand, the analysis carried out on the grains was found within the values reported by other authors.

## 1. Introduction

For the Mexican population, maize constitutes the main source of food, with important historical roots. In particular, in the Apan plains region, located southeast of the state of Hidalgo, maize is grown between 2360 and 2468 masl, under rainfed conditions and on soils with reduced availability of macronutrients [1], conditions that predispose a certain vulnerability to adverse weather conditions [2]. In order to obtain a good harvest yield, it is necessary to consider sustainability in production and, in this sense, the loss of soil fertility is the main concern of farmers [3]. In this regard, the most important adverse conditions are high humidity, low temperatures, diseases and poor nutrient assimilation by plants [4]. These conditions are caused mainly by the reduction in the organic matter content of the soil together with unbalanced fertilization, as a result of bad agronomic practices derived from modern agriculture [5], where the soil microbial activity and the biochemical cycles of nutrients play a very important role in maintaining the fertility of agricultural soils [6].

Because of a preliminary evaluation carried out in the Apan plains region, the reduction in soil fertility, dependence on non-renewable resources and degradation in the composition of the edaphic ecosystem were identified as limiting factors, due to the application of synthetic agrochemicals, such as fertilizers and pesticides [7]. In this sense, about half of nitrogen-based synthetic fertilizers are lost in the environment, contaminating water, soil and the atmosphere [8]. In addition, several studies suggest the substitution of chemical fertilizers for the use of biofertilizers, also known as bioinoculants [6]. These bioinoculants allow for low-cost production without environmental contamination, improving soil fertility and biodiversity [9]. Among all the endophytic microorganisms that are used as biofertilizers (bacteria and fungi), fungi of the genus *Trichoderma* stand out for their different modes of action through which they can interact with plants [10], in addition to the role they play in the bioregulation of pathogenic microorganisms associated with crops [11].

For corn and other grasses, the use of chemical fertilizers, such as nitrogen (N), phosphorus (P) and potassium (K) is essential to increase their yield [12]. However, its production and application consume fossil fuels and adds a large amount of CO_2_ to the atmosphere [13]. On the other hand, its use efficiency ranges between 10–60%, depending on the type of fertilizer, crop and production system [14]. Therefore, most of these fertilizers are environmental pollutants with a great impact on ecosystems and human beings [15], causing eutrophication of aquatic systems and water wells used for human consumption and irrigation [16]. In addition, excessive fertilization favors the leaching of nitrates into the underground aquifer, which has generated an increase in nitrogenous compounds, which can become pollutants, such as nitrosamines, precursors of cancer in humans [17]. Faced with this situation, the current trend is to significantly reduce the use of these synthetic agrochemicals in agriculture, replacing them with bioinoculants that increase crop yields, improve soil fertility and reduce populations of phytopathogenic microorganisms. This, therefore, minimizes the costs of synthetic inputs and environmental pollution, thus, contributing to the sustainability of agriculture. Consequently, the presence of endophytic fungi of the genus *Trichoderma* in the roots will benefit corn plants, stimulating their defense systems and increasing their competitiveness and yield [18]. In this sense, the variety of purple corn of the Kculli race stands out, due to its importance as a substitute for synthetic dyes in food [19] and in the manufacture of pharmaceutical and cosmetic products [20]. As well as its positive effects on health, due to its nutraceutical and antioxidant properties [21], it is able to prevent some types of cancer [22]. Therefore, the aims of the study were as follows: (i) to evaluate the effect of different fertilization schemes on the agronomic performance of purple maize plants of the Kculli race, and (ii) to evaluate the main chemical characteristics of the purple maize grains produced from the different fertilization schemes evaluated in corn plants.

## 2. Materials and Methods

### 2.1. Fungal Cultures

*Trichoderma harzianum* strains (JCP1 and JCP2) used in this study were isolated from the aerial tissue of maize plants of the conical race, grown in the municipality of Apan, Hidalgo, Mexico, during the spring–summer crop cycle of 2018. For the isolation of the fungal strains, 5 mm fragments of aerial tissue from corn plants were sterilized for 1 min in a 3% sodium hypochlorite solution and rinsed twice in sterile distilled water. Subsequently, the plant tissue fragments were dried on filter paper and placed in Petri dishes with a potato dextrose agar medium (PDA, BD Bioxon^®^) in a humid chamber and incubated in the dark at 25 °C for 7 days [23]. The strains were maintained in a PDA medium, at −4 °C and as a spore suspension at −87 °C.

### 2.2. Molecular and Morphological Characterization

DNA extraction of JCP1 and JCP2 strains was performed as described by Cuervo-Parra et al. [24], where a region of nuclear DNA from each strain (containing the ITS sequence) was amplified by PCR, using the primers ITS5 and ITS4B [25]. The PCR amplifications were performed as described by Romero-Cortes et al. [26]. Pairwise sequence alignments were performed with the online BLAST program, with sequences from the NCBI database [27]. The phylogenetic analysis of the sequences of strains JCP1 and JCP2 and other 21 related NCBI sequences was performed by building a phylogenetic tree using the statistical method Neighbor-joining, the Jukes–Cantor model, supported by the bootstrap method of a phylogeny test, with 1000 random replicates through MEGA X software [28]. Morphological descriptions were based on the comparisons with other publications of *T. harzianum* [29,30,31] augmented by new observations. For morphological identification, a PDA medium was used. The macroscopic characterization of the colonies was carried out by recording the morphology, color, growth rate and release of pigments into the medium of each colony. For the microscopic description, 50 measurements of the following structures were made: conidia, conidiophores, phialides and chlamydospores. The measurements of the mentioned characteristics were obtained from images taken using the IOS 14.7.1 software, a 12-megapixel single camera system, a wide-angle camera with ƒ/1.8 aperture on an iPhone SE (Model MX9U2LZ/A, Mexico City, Mexico). Scanning electron microscope micrographs were taken with the Cry-transfer system (SEM; JEOL, Model IT300, Boston, MA, USA) at the Escuela Superior de Apan (ESAp-UAEH). Subsequently, all photographs were digitally captured using CorelDraw 2017 software (CorelDRAW Graphics Suite, version 2017, Mexico City, Mexico).

### 2.3. Corn Accessions

For this study, the Kculli maize race was used, defined within the group of primitive races, which is cultivated in various geographical regions of Peru, Bolivia, Argentina, Brazil and Mexico, normally between 500 and 4000 masl, in deep soils without much moisture retention [20,32]. For seed multiplication, seeds of the CIMMYTMA 12,580 and CIMMYTMA 12,473 accessions were used [33,34] from the CIMMYT germplasm bank [35].

### 2.4. Treatments and Experimental Design

A preliminary experimental plot of 403.2 m^2^ was carried out on March 1 2018 under temporary conditions, in the municipality of Mineral de la Reforma, Hidalgo, Mexico, with the geographic coordinates 20°07′48″ North latitude, 98°73′28″ West latitude, at 2426 m altitude, to test the effect of different fertilization schemes on maize plants of the Kculli race. A completely randomized design with three replications for each treatment was used. Each experimental treatment consisted of 4 furrows 6 m long, separated by 0.8 m. A seed was sown every 25 cm, which represented a population density of 40,000 plants ha^−1^. Per treatment and repetition, 280 plants were used, where each useful experimental treatment consisted of the two central rows, with a maximum number of 48 maize plants, to avoid cross-contamination and the edge effect [36]. In total, 2016 seeds distributed among the seven treatments evaluated were used. For agronomic management of the crop, we applied the recommendations for high-yielding maize [37]. Based on the results obtained in a sample of 30 plants per repetition and 90 plants per treatment from the preliminary field experiment (data not shown), the experimental design was generated to evaluate the different fertilization schemes in purple corn plants to be tested during the 2019 crop cycle (Table 1). The grain yield was not estimated because the plants were used to multiply the grain, and the harvest and shelling were done manually.

The field experiments were carried out in 2019 under temporal conditions, with an average annual temperature of 14.4 °C and a total annual rainfall of 618.2 mm, at the facilities of the Escuela Superior de Apan, located at the following coordinates of 19°39′20″ North latitude, 98°31′05″ West latitude and at an altitude of 2488 masl, in the state of Hidalgo [38,39]. The total area of the experimental plot was 672 m^2^ (20 m wide by 33.6 m long), where treatments were distributed using a completely randomized design, with three replications for each treatment. A space of 1 m without sowing was left between repetitions planted on the same furrow along the width of the plot. The characteristics of each repetition consisted of 6 rows of 6 m in length, with a separation between the rows of 0.8 m. For each row, a total of 48 seeds were sown, distributed in 24 holes, placing 2 seeds per hole, with a separation of 25 cm between seeds. Subsequently, after 51 days of vegetative growth, one plant per hole was thinned, leaving the most vigorous plants to carry out the morphological characterization of the maize plants of each treatment. Each useful experimental treatment consisted of the four central rows, with a maximum number of 96 maize plants, to avoid cross-contamination between treatments and the edge effect between repetitions [36,40].

Soil preparation consisted of a fallow and a cross. Based on the report of annual precipitation (mm) reported for the study area [39], the temporality in which the sowing of the experimental plot was carried out was in the period of time between the months of April–September of the year 2019. An experimental design was used to evaluate the effect of the different fertilization schemes tested in corn plants (Table 1). The treatment T1 did not include the application of any type of fertilizer; treatment T2 included a dose of N (0.6048 kg), P_2_O_5_ (0.24192 kg), K_2_O (0.504 kg), MgO (0.0288 kg), S (0.10656 kg), Zn (0.0072 kg) and B (0.00576 kg). Treatment T4 included a dose of bioinoculants of (0.001152 kg), based on zeolite as an inert ingredient at 80% (0.6144 g), and 2 × 10^7^ CFU of a mixture of the strains JCP1 and JCP2 of *T. harzianum* as an active ingredient at 20% (0.1536 g). The mixture of zeolite and bioinoculants was applied directly on the rhizosphere of the corn plants in the aforementioned dose.

The treatment T5 included a dose of sphagnum peat at 25.83% (31 kg); 25% solid earthworm humus (30 kg); 3.33% ground mesquite charcoal (4 kg); ground coffee bagasse at 16.67% (20 kg); and ground alfalfa hay at 29.17% (35 kg). Finally, for treatments T3, T6 and T7, the same number of materials described for the individual treatments (T2, T4 and T5) was used in the specified combination. The composition of the aforementioned materials used for all treatments and repetitions was adjusted to the dose required for an area of 28.8 m^2^. The treatments were managed in the same way throughout the crop cycle. After this period of time, the growth and yield variables of the corn plants per treatment were measured. The different fertilization schemes at the doses mentioned above were carried out in three stages during the vegetative crop development at 30, 60 and 90 days previous to male flowering.

### 2.5. Variable Definition

In this research, the effect of different fertilization schemes on the growth and yield of purple maize plants of the Kculli race was evaluated through a randomized block design. The characteristics of growth and yield during the different stages of development of the maize plant were defined as the necessary parameters to observe the changes in the variable of time on the growth (stages of vegetative development and flowering) and yield (stage of maturation) of maize plants.

#### 2.5.1. Independent Variables

For time, it was proposed from the beginning when the maize seeds were sown in the experimental plots in the month of April, until the harvest stage at the end of the crop cycle in the month of September (183 days).

#### 2.5.2. Growth Dependent Variables

For plant height and stem diameter, the maximum height of the plant, including the panicle, was measured. The height was calculated by measuring from the soil surface to the apex of the spike in centimeters and the diameter of the stem was measured at 5 cm above the ground. For the days to male and female flowering, this was quantified in days from sowing until 50% of the selected plants of each useful experimental treatment released pollen from the panicle and exposed their stigmas on the ears. Subsequently, the floral asynchrony (FA) was calculated using the following formula: FA = days 50% FF—days 50% FM, where FA represents the interval between the emission of the pistils in the ear and anthesis in the panicle; FF is the time at which 50% of the plants have exposed their silks on the ear; and FM is the time at which 50% of the plants have their panicles exposed and have begun to shed pollen [41].

#### 2.5.3. Yield Dependent Variables

For the number of ears per plant, the total number of ears was quantified and divided by the total number of plants for each useful experimental treatment. For ear length, the distance in cm from the base to the apex was measured. Subsequently, the ear diameter, in cm, was calculated with a vernier, measuring the middle part of the upper ear. The weight of the ear was determined by calculating the total weight of each ear in grams. Likewise, the number of grain rows was calculated for the same ears, quantifying the number of rows in the middle part of the upper ear. The number of kernels per row was measured from base to apex on the top ear. The grain width and length were measured with a vernier, taking the measure of 100 grains, making the measurement in cm. The weight of 100 grains was quantified by taking 100 grains at random and their weight was determined [42]. The measurements to determine the weight of the ear and the grains were made on a pomegranate scale (DIDIHOU Brand, electronic coffee scale model K1802, USA) with a maximum capacity of 3 kg. Finally, the yield of each treatment adjusted to 12% H_2_O was calculated using the conversion factor of the harvested area with respect to one hectare, expressed in t ha^−1^ [43].

### 2.6. Corn Starch Isolation

The starch was isolated according to the methodology proposed by Sandhu et al. [44] and Bustillos-Rodríguez et al. [45]. Corn kernels were finely ground using a food-grade stainless steel high-speed electric mill (Semillas de Vida, Model SV-MO-100T, Cuernavaca, Morelos, Mexico). Per sample, 1 kg of ground corn was mixed with 2 L of 0.1% NaOH and kept at 4 °C for 18 h. The mixture obtained was homogenized at maximum speed in a stainless-steel industrial blender (TAPISA^®^, model T5L, Mexico City, Mexico). Subsequently, each sample was passed through 100 and 270 mesh sieves (Advantech Manufacturing, Inc., New Berlin, WI, USA), with a pore size of 150 and 53 µm, respectively, allowing the suspension to settle for 18 h. The supernatant was then discarded and the retained solids were washed three times to remove NaOH. The supernatant elimination process was carried out by centrifugation at 4153× *g*, 10 min, using a centrifuge (Hettich^®^, model EBA 21, Tuttlingen, Germany). The starch obtained was placed in cylindrical plastic trays (16 × 13 × 6 cm) and dried at 40 °C for 24 h. Finally, the starch was ground in a mortar, passed through a 100-mesh sieve and stored in sealed plastic bags at 25 °C [45].

### 2.7. Proximal Analysis of Starches

The proximal analysis of the starches was carried out according to the official AOAC methods 923.03, 920.35, 925.10 and 991.20 [46] to determine the ash, fat, moisture and protein content (N × 6.25), respectively.

### 2.8. Fourier Transform Infrared Measurement

An analysis by Fourier transform infrared (FTIR) spectra of the starches was performed, following the methodology described by Zamudio-Flores et al. [47]. The FTIR spectra were recorded from 500 to 4000 cm^−1^ with a resolution of 4 cm^−1^ for 34 scans using a spectrophotometer (NicoleTM iS10, Thermo Scientific, Waltham, MA, USA), equipped with a universal attenuated total reflectance accessory (ATR).

### 2.9. Tristimulus Color

The starch color was determined using a colorimetry meter (Konica Minolta Sensing Americas, Inc., model CR-400, Tokyo, Japan) with diffuse illumination geometry, a 0° viewing angle, and an 8 mm port/viewing area. Prior to starch evaluation, the colorimeter was calibrated with a standard blank. Each starch sample (random) was evaluated five times (L*, a* and b*).

### 2.10. Statistic Analysis

The analyses to measure the growth variables during the vegetative development and flowering stage were carried out in the months of May–June and June–August, respectively. The analyses for the yield during the stage of grain filling and maturity were carried out between the months of August–September. Sánchez et al. [48] proposes the most appropriate characters for the classification of the different groups of maize, which are also useful to be able to evaluate the effect of the different fertilization schemes on the variables of growth and yield of the maize plants studied. The results were compared using a completely randomized design with three replications, through ANOVA analysis and the Tukey HSD all-pairwise test for the comparison of means, with a significant difference of (*p* ≤ 0.05). All statistical analyses were performed using Statisticx 10 software [49].

## 3. Results and Discussion

### 3.1. Morphological Characterization

**Figure 1:**
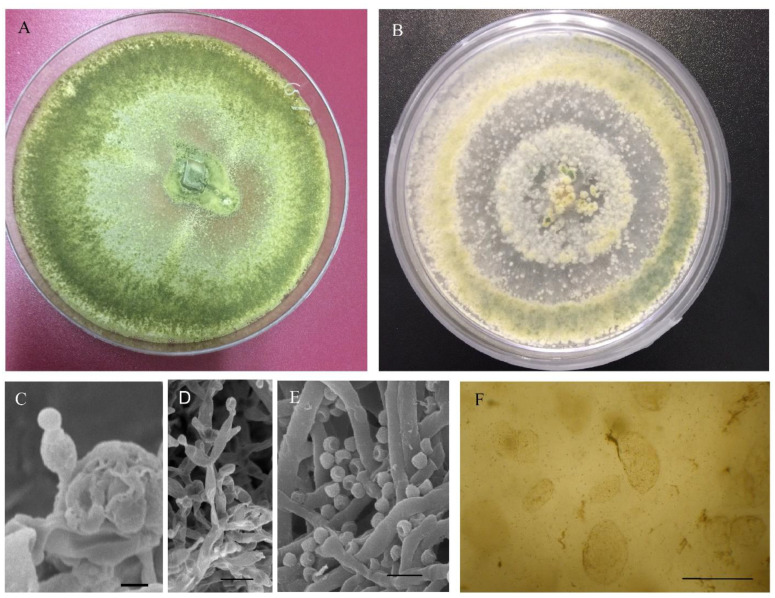
Colonies of *T. harzianum* in a PDA medium (5 d, 25 °C). Obverse of plate (**A**,**B**); SEM micrographs of phialides (**C**), bar = 2 µm, phialides and conidia (**D**), bar = 5 µm and conidia (**E**), bar = 5 µm; and chlamydospores (**F**), bar = 10 µm.

The colonies of the JCP1 and JCP2 strains grew and matured rapidly after five days of incubation in a PDA culture medium, showing a diameter of 78.5 to 80 mm and an average 79.58 ± 0.11, 15.91 mm/day, data that agree with what was previously reported for other isolates of *T. harzianum* [50,51]. The mycelium at first was scarce and hyaline, and over time it acquired a cottony appearance and an olive green to yellowish green color, due to the production of spores in conidiogenous pustules, without the release of pigments into the medium, characteristics that coincide with the reports of other isolates of *T. harzianum* isolated from Ecuador [30]. The pustules were cotton-like in texture, distributed throughout the medium or in concentric rings from the center of the culture medium (Figure 1A,B). Since no significant differences were observed between the values calculated for the morphological structures of the two strains, the average values of the morphological structures were calculated, which are described below. The hyaline hyphae presented highly branched conidiophores, which were not whorled, 62–69.5 µm long and 2.9–5.7 µm wide, (mean values: 64.82 ± 2.15 µm long, confidence interval (CI) = 63.49–66.14, *n* = 50; and 4.83 ± 0.53 µm wide, CI = 4.5–5.16, *n* = 50), with individual or grouped phialides, strongly constricted at their base (Figure 1C,D), 6–16.2 µm long and 2- 4.6 µm wide (mean values: 8.85 ± 3.71 µm long, CI = 6.57–11.13, *n* = 50; and 3.35 ± 0.75 µm wide, CI = 2.89–3.82, *n* = 50).

These results are within the values previously reported for the strains of *T. harzianum* isolated from a horticultural area of the Toluca valley [29]. The conidia were subglobose, ovoid or ellipsoidal in shape, 3–8.6 µm long and 3–4.7 µm wide, (mean values: 4.5 ± 1.65 µm long, CI = 3.49–5.52, *n* = 50; and 3.95 ± 0.38 µm wide, CI = 3.72–4.19, *n* = 50) and arranged in terminal whorls of up to 4 conidia (Figure 1E). The chlamydospores were intercalated individually, formed in the submerged mycelium (Figure 1F), although sometimes two or more chlamydospores may fuse [50], subglobose of 6–14.3 µm long, and 10.0–14.3 µm wide (mean values: 10.01 ± 0.97 µm long, CI = 9.07–10.95, *n* = 50; and 12.54 ± 12.54 µm wide, CI = 11.94–13.14, *n* = 50). Based on phenotypic characteristics, radial growth at 25 °C for 5 days, and measurements of microscopic structures, the two strains were identified as *T. harzianum* [18,31].

### 3.2. Molecular Characterization

**Figure 2:**
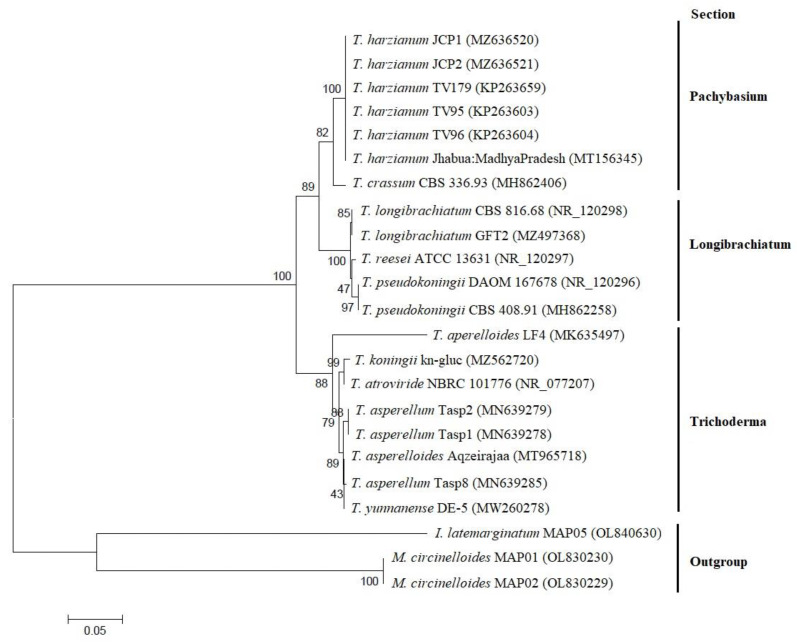
Phylogenetic tree for *Trichoderma* strains JCP1 and JCP2, generated with the Neighbor-joining method, obtained by analyzing the Jukes–Cantor model based on ITS sequence data of *Trichoderma* species from NCBI GenBank. Adjacent numbers in nodes are bootstrap values and accession numbers are indicated in parentheses.

The BLAST analysis confirmed the previous morphological identification of strains JCP1 and JCP2, placing the two isolates within the section Pachybasium in the *T. harzianum* group. The ITS sequences of strains JCP1 and JCP2 were deposited in the GenBank database under the accession numbers MZ636520 and MZ636521, respectively. The 23 sequences selected for the multiple sequence alignment ranged from 498 to 665 bp in length, with a consensus region length of 715 bp. The phylogenetic analysis generated a tree by the statistical method Neighbor-joining, which grouped the sequences of the JCP1 and JCP2 strains with *T. harzianum* sequences, isolated from maize fields [51] and corn plants [52], with a bootstrap statistical support value of 100 and an identity percentage of 99.83 with the BLAST program [53]. In order to establish phylogenetic relationships between the sequences used, the phylogenetic tree was rooted with sequences from *Irpex latemarginatus* (OL840630) and *Mucor circinelloides* (OL830229, OL830230) from GenBank as an outgroup [25]. The best tree with the sum of the length of its branches = 1.15472297 is shown below (Figure 2).

### 3.3. Growth Dependent Variables

#### 3.3.1. Plant Height

Once the maize plants reached their maximum height with the flag leaf visible, and their panicle fully developed, the maximum plant height was measured. The time lapse varied significantly between treatments, from 96 to 116 days. The best results were obtained in treatments T7, T4 and T6, with a total plant height of 315.34, 283.46, and 275.43 cm, respectively (Table 2). In this regard, the combined application of biofertilizers, enriched compost and recommended doses of N, P and K results in the highest growth of corn plants [54,55,56]. In this sense, in purple corn plants of the varieties PVM-581 and INIA-615 from Peru, plant heights of 280 and 288.1 cm have been obtained [57]. Therefore, the supply of N that is provided to the crop will have a considerable influence on the maximum height that the plants can reach [12]. In addition, the simultaneous interaction of the bioinoculants added with the soil’s own microorganisms can potentiate the benefits of the inoculants for the plants [58].

The fungi of the genus *Trichoderma* stand out because they favor the growth of plants [59] through the mineralization of organic matter, the solubilization of orthophosphates, phyllosilicates, the control of pathogens, as well as the transport of nutrients and water [60], which is reflected in the increased development of the root system and better assimilation of nutrients [61]. On the other hand, in treatments T3, T5 and T2, lower values of 263.33, 262.7 and 253.3 cm of plant height were recorded (Table 2), respectively. Slightly lower results, between 195 and 215 cm, were reported for the other improved purple maize varieties in Peru [62]. In addition, the plant heights reported for the treatments T3, T5 and T2 were higher than the plant height of 207 cm obtained for the AYA-77 maize accession of the Kculli race in a previous study [19]. In this regard, maize plants of the Kculli race, cultivated under an adequate scheme of chemical fertilization, irrigation and contribution of organic matter, can reach up to 4 m [20]. These are heights that, due to various biotic and abiotic factors to which the maize plants are exposed, they do not usually reach [36,63,64]. In addition, the fertilizer dose, the genotype used and the harvest year are other factors that influence the adequate use of N, P and K fertilizer by maize plants [65].

Other authors report increases in plant height and yield when the crop was fertilized with farmyard manure and NPS inorganic fertilizer, at a dose of 245.1 kg ha^−1^ [66]. In this sense, the presence of organic matter in the soil and its degradation by microorganisms allowed for adequate nutritional and moisture retention conditions, which is reflected in the height reached by the plants of treatments T7, T6 and T5. In this regard, it was reported that, for the roots of the corn plants that were not inoculated with microorganisms, a root colonization of 7.83% was observed from native soil mycorrhization [67]. Organic fertilizers provide organic matter, nutrients and additional microorganisms that favor soil fertility and the subsequent nutrition of crops [68]. Therefore, the contribution of organic fertilizers, based on manure, to the corn crop result in increases in plant height [69]. The bioinoculants are those responsible for increasing the availability of nutrients, as N, P, Fe and Zn do for plants and/or provide new nutrients to the soil-plant system, as well as promote the growth and production of the corn crop [70,71]. In this regard, soil management is important to promote the retention of organic matter and adequate conservation of soil moisture [72]. However, the capacity of the organic fertilizers as a source of nutrients for plants is low, compared to chemical fertilizers and/or bioinoculants [61], as was observed in this research.

Finally, regarding treatment T1, the lowest plant height with an average value of 185.63 cm (Table 2) was observed, a result that is within the plant height range of 192.41 to 151.13 cm and was reported for two accessions of maize of the Kculli race grown in Peru without nutrient input [73]. In this sense, this race under rainfed conditions and without the addition of fertilizers presents small plants barely between 92 to 176 cm tall, without tillers and with around 10 leaves [74,75]. In this regard, the lack of nutrients in the soil of the treatment T1 plants resulted in the decrease in the recorded plant height, since N is an essential structural and metabolic component of the plant cell, and P is important during cell division and the development of new plant tissue [76].

#### 3.3.2. Stem Diameter

The highest values were recorded in treatments T7 and T2, where the plants reached 2.06 and 2.02 cm of stem diameter, respectively. It should be noted that, in these treatments, the plants had all the nutrients required to develop well, which is directly related to the height values recorded for these two treatments (Table 2). These results are similar to those obtained for the other plants of the Kculli race cultivated in Peru, with stem diameters of 2.05 to 2.27 cm [73]. In this regard, bioinoculants together with the contribution of chemical fertilizer result in an increase in the plant stem diameter [40,67]. Slightly lower results were recorded in treatments T3, T4, T5 and T6 with values of 1.94, 1.89, 1.87 and 1.83 cm of stem diameter, respectively. A beneficial effect has been found on the growth of the shoots and roots, as well as on the root neck and stem diameter of maize plants when they were inoculated with *T. harzianum* strains [77]. Finally, the smallest stem diameter occurred in treatment T1, where the plants acquired an average value of 1.30 cm, due to nutrient deficiency and/or lack of interaction with microorganisms in the soil (Table 2).

#### 3.3.3. Days to Male and Female Flowering

The analyses carried out for the variables of days to 50% of male and female flowering showed significant differences between the different fertilization schemes evaluated (*p* ≤ 0.05). Treatments T7 and T3 were the earliest, with average values of 98.67 and 100.67 days for male flowering, and from 103.33 and 106.33 days for female flowering, respectively. Shorter time intervals for male flowering, from 83 to 96 days, and female flowering, from 90 to 102 days, have been reported in other studies with other varieties of purple corn planted in Peru [78,79,80] and Mexico [19]. In this regard, a decrease in the flowering time of corn plants has been reported when they were inoculated with bioinoculants and chemical fertilizer [81]. Because these improved purple corn varieties are derived from the ancestral Kculli purple corn line [78], the results obtained in this study are relevant, since they are at the level of the values reported for the above-mentioned improved purple corn varieties. Since the number of days to flowering reported for the Kculli purple corn in Peru is between 100 and 120 days [82], even reaching extremes of 140 and 144 days [83], the earliness observed in the purple corn samples evaluated in this study is relevant. On the other hand, treatments T5 and T6 were statistically similar in their flowering, reaching 111 and 105.33 days for their male flowering, and an average of 118.33 and 112.67 days for their female flowering, respectively (Table 3).

Time intervals from male and female flowering of 106 and 111 days were reported in the plants of the Kculli race grown in Cusco, Peru [73], results that are similar to those obtained for treatment T6 in this study. With respect to treatments T2 and T4, average values between 113.67 and 115.33 days for male flowering and 122 and 123.67 days for female flowering were observed, respectively. In this regard, several studies show the beneficial role of bioinoculants [40,61] and chemical fertilizers [79,84] on corn yield, where bioinoculants improve soil fertility, decompose organic matter and make it possible for plants to absorb the nutrients necessary for their growth and development [85]. Lastly, treatment T1 was the latest to flower, with 118.67 days for male flowering and 128.67 days for female flowering (Table 3). Similar results for male and female flowering of 122 and 128 days were reported for plants of the Kculli race, grown without nutrient input in Peru [73].

#### 3.3.4. Floral Asynchrony and Physiological Maturity

Derived from the statistical analysis, the differences found for floral asynchrony (FA), were highly significant between treatments, where treatments T7 and T3 presented the lowest FA with 4.67 and 5.67 days of difference, respectively, and treatment T1 showed the highest FA with a time of 10 days. Likewise, the other treatments showed intermediate values, with FA times of 7.33 days for treatments T6 and T5 and 8.33 days for treatments T2 and T4. In this regard, several improved varieties of purple corn, grown in Peru, reported FA values between 4.8 to 5.9 days [80], which are similar to those reported in treatments T7 and T3 (Table 3). Likewise, it has been shown that the release of pollen and the emergence of the stigmas occurs discontinuously during a short period of approximately five to eight days, under favorable temperature and humidity conditions. Where the lifespan of a pollen grain is approximately 20 min after it is shed and if an ovule is not successfully fertilized within that period, the stigma dies without being fertilized and the part of the ear that it is attached to becomes sterile [86]. Therefore, at higher FA observed in a corn crop, the number of sterile ovules per ear will increase, which will have a negative impact on the grain yield of the plot.

On the other hand, the time elapsed between flowering and physiological maturity of the treatments considered in this study was between 61 and 65 days. These values are within the range of 66 to 78 days, which was reported for the other improved purple corn varieties grown in Peru [78,87]. In addition, the total time from germination and seedling emergence to physiological maturity of the crop varied significantly between treatments, from 147.67 to 180.67 days. In this regard, longer times to physiological maturity of between 213 and 229 days were reported for purple corn plants of Kculli race grown in Cusco, Peru [73]. Shorter harvest times of four months from sowing to physiological maturity have been reported for improved purple maize of the INIA-601 variety, grown at 18 masl [82]. In this sense, other authors have mentioned that the altitude at which a variety of purple corn is planted will affect the time in which the crop will reach its physiological maturity [88]. It was found that the altitude of the study area (2488 masl) did not affect the time in which the plants of the different treatments reached their physiological maturity of between 4.92 and 5.63 months, with the exception of treatment T1 (Table 3). Data that are consistent with the physiological maturity times of 5.67 and 5.96 months were reported for several improved purple corn varieties [78,80,87].

Finally, in relation to the longest time to physiological maturity, which was recorded for treatment T1, when corn plants are under stress conditions due to a lack of water, nutrients, light, and/or high temperature, the emission of pistils and the growth of the ears stops [89]. In addition, increasing the FA causes problems in pollination and grain filling in the ear [80], which would explain the result obtained in treatment T1, where the general deficiency of nutrients caused a delay in growth, plant height, as well as late maturity and the low grain yield observed.

### 3.4. Yield Dependent Variables

#### 3.4.1. Number of Ears per Plant

A significant effect of the distance between plants has been reported on the number of ears recorded per plant, with averages of 1.11, 1.06 and 1.05 ears per plant for distances between plants of 30, 20 and 25 cm [90], respectively. These results are similar to the values recorded in treatments T7, T2 and T3 for the number of ears per plant, with values ranging from 1.16 to 1.19 ears per plant (Table 4).

**Figure 3:**
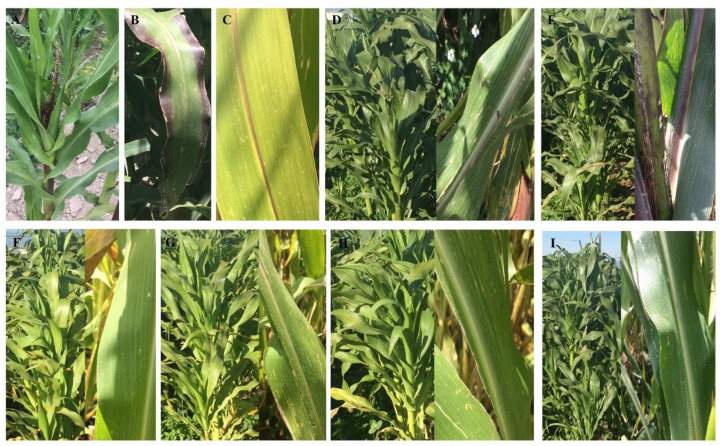
Purple corn plants of the Kculli race, at 103 days of growth. Treatment T1, plants with light green leaves, with white interveinal chlorosis that progresses from the tip to the base of the leaves, due to a deficiency of Mg (**A**); leaf with a coloration that goes from light green in the center to purple on the edges, due to a deficiency of P (**B**); light green leaf due to N and K deficiency (**C**); treatment T2, plants with dark green leaf coloration (**D**); treatment T3, plants with dark green growth and leaf coloration, without symptoms of nutrient deficiency (**E**); treatment T4, plants with light green leaf coloration and with new leaves with whitish yellow interveinal chlorosis, due to K and Zn deficiency (**F**); treatment T5, plants with light green leaves, with light yellow chlorosis in the old leaves, due to K deficiency (**G**); treatment T6, plants with a general loss of the healthy dark green color of the foliage, due to lack of K (**H**); and treatment T7, plants with adequate development and dark green leaf color, without signs of macro and micronutrient deficiency (**I**).

Higher values of 1.43 ears per plant were recorded in the INIA-601 variety of purple corn, cultivated under a chemical fertilization scheme [79]. Lower values of 1.2 ears per plant for the starchy maize plants of the INIA-606 variety was reported [75]. On the other hand, for treatments T1, T4, T5 and T6, the number of ears per plant was lower, with values between 0.7 and 0.96. This result was due in part to the number of plants without ears that were recorded in these treatments and to the nutrient deficiency observed in the leaves of these maize plants (Figure 3), which had a negative impact on the number of ears obtained. In another study conducted with corn plants of the Kculli race grown in Cusco, Peru without the addition of fertilizer throughout the crop cycle, an average of one ear per plant was reported [73].

#### 3.4.2. Ear Weight

The weight of the ear is an important variable of the yield in the corn crop. This was evidenced in the ear weights obtained in the treatments evaluated in this study, registering the highest ear weights in treatments T7 and T2, with values of 96.62 and 85.18 g, respectively. In this regard, for the plants of the Kculli race grown in Cusco, Peru, ear weight values between 86.79 and 98.82 g were reported [73]. Likewise, an ear weight of between 90.66 and 126.34 g for the purple corn of the variety PMV-581 was recorded [91]. Although, some ears weighing between 101.6 and 149.4 g were recorded in this research the average weight of ear per treatment was lower (Table 4).

**Figure 4:**
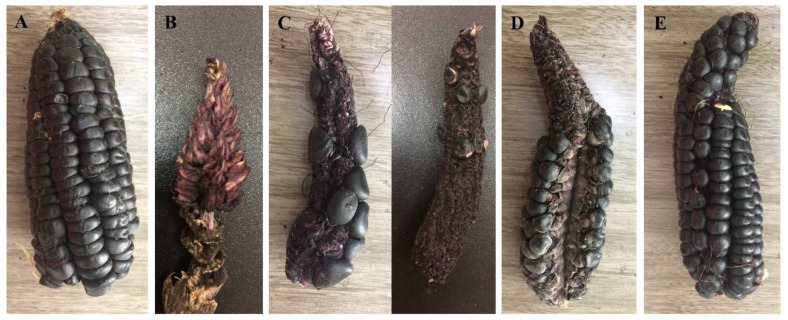
Sample of ears of corn from the Kculli race of treatment T1, with different signs of nutrient deficiency. Small ear with small grains, due to Mg and Mn deficiency (**A**); aborted ear without any grain formed (**B**); poorly filled ears with very few grains formed, due to N and K deficiency (**C**); small ear with P deficiency, with crooked tip and poorly developed kernels (**D**); and small ear, crooked at the tip due to grain abortion (**E**).

On the other hand, in treatments T6, T5 and T3, values of 74.19, 72.91 and 71.63 g were recorded for the average weight of the ear, respectively. In this regard, in purple corn plants of the Canteño variety, managed under a fertilization scheme based on organic matter and chemical fertilizers, ear weights of between 77.89 to 97.52 g were reported [62]. Higher ear weights, between 86 and 232.25 g per ear, have been reported for other improved purple corn varieties, managed under a chemical fertilization scheme [57,88,91,92]. Finally, for corn plants of treatments T4 and T1, values of 61.44 and 25.15 g were recorded for the weight of the ear; in addition, a high number of ears with aborted grains and/or without grains was found in treatment T1 (Figure 4), a situation that had a significant impact on the grain yield recorded in this treatment (Table 4).

#### 3.4.3. Grain Yield

The final yield of the corn crop is the result of the following two simultaneous and interdependent processes: the growth and development that leads to the establishment of the morphology of the adult organism, as the ontogenic cycle progresses [93]. In this sense, the grain yield observed in this study varied significantly among the treatments evaluated, where the best results were recorded in treatments T7 and T2, with values of 6.19 and 4.35 t ha^−1^ (Table 4). Superior results with yields of 7.78, 7.97 and 8.22 t ha^−1^ were reported for purple corn plants of the Canteño variety, fertilized with organic matter, bioinoculants and organic matter and bioinoculants and potassium chloride [94]. It is evident that the best result reported in this work was obtained when the three types of fertilizers were combined. Furthermore, the yield difference observed between the treatment T7 and grain yield of 8.22 t ha^−1^ reported in the previous work may be due to the fact that the variety used in the aforementioned work corresponds to an improved maize, which is why it is logical that its grain yield was higher than expected for the Kculli race, which is the ancestral maize race from which all the improved varieties of purple maize currently grown were developed [78]. On the other hand, regarding the application of chemical fertilizers, several studies report grain yields between 4 to 4.8 t ha^−1^ for different varieties of improved purple corn plants [79,90]. These results are similar to those recorded in this work for treatments T7 and T2 that included chemical fertilizer within their formulation. Lower grain yields between 2.78 to 3.67 t ha^−1^ for the purple corn plants of the Kculli race, including the INIA-615 and PMV-581 varieties, fertilized with chemical fertilizers, were reported [73,95,96], results that are lower than the grain yield obtained in treatment T2 (Table 4). Higher yields of 11.8 t ha^−1^ for the purple maize plants of the INIA-601 variety grown in Peru were reported [80]. In this sense, the heterogeneity observed in these results is mainly related to the difference in altitude, soil composition, temperature, precipitation during the crop cycle, maize variety and the experimental design used [97].

On the other hand, grain yields higher than 3 t ha^−1^ were obtained in treatments T3, T6, T5 and T4 (Table 4). In this sense, there are reports regarding the increase in yield and quality of fruit derived from the inoculation of a crop with bioinoculants [56,61,98]. These results are consistent with the grain yield obtained in the corn plants of the treatments T3, T4, T6 and T7 that included bioinoculants within their formulation. This is due to the solubilization of soil macronutrients by bioinoculants, with which there is a greater availability of nutrients for crops that have been inoculated with microorganisms. In another study carried out with hybrid maize P3258W from Pioneer, they found that the manure-based fertilizer increased crop yield by 9.6%, compared to the other fertilization schemes evaluated [6], a result that is consistent with the yield increases observed in treatments T5, T6 and T7. However, the grain yield results obtained in treatments T5 and T6 were lower, compared to the results obtained in the treatments that included chemical fertilizer and/or bioinoculants (Table 4). In addition, symptoms of K and Zn deficiencies were observed in the plants of these treatments (Figure 3). In this regard, it was reported that the application of high doses of organic fertilizer (30 t ha^−1^) in hybrid maize plants was not enough to produce grain yields higher than the yields obtained with the conventional chemical fertilization treatment evaluated [99]. It is evident that the level of fertilization and water applied to a crop will largely determine the accumulation of biomass and the harvest index and yield that the plants of that crop can achieve [100]. In this sense, the lack of the main nutrients N, P and K in the soil constitutes the main cause of the reduction in the grain yield of corn crops, reaching yields of less than 1 t ha^−1^ [101]. This explains the lowest yield of 1.02 t ha^−1^ recorded in treatment T1, where not one fertilization scheme was applied to the maize plants of the Kculli race. In this regard, in purple corn plants cultivated in a traditional way by farmers in the Sierra of Peru, an average yield of 1.5 t ha^−1^ is reported [80]. However, the lack of economic solvency of many producers who plant corn under rainfed conditions does not allow them to pay for the high production costs involved in the use of chemical fertilizers; consequently, the low yields they obtain result in a limited profit margin.

#### 3.4.4. Ear Length

From the statistical analysis, the differences found for the length of the ear between the treatments varied significantly (*p* ≤ 0.05), observing the best results in treatments T7, T6 and T2 with ear lengths of 18.95, 17.25 and 17.2 cm (Table 5), respectively. Similar values of ear length between 12.15 and 21.11 cm, corresponding to the genetic materials CMC-026 and CMC-212 of purple maize of the Kculli race, cultivated in Cusco, Peru were recorded [73].

**Figure 5:**
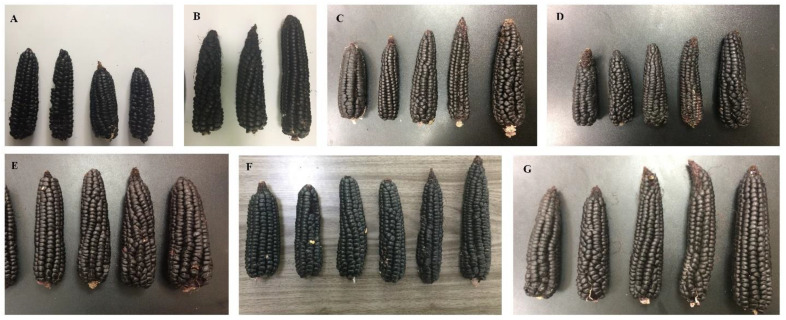
Photographs of the ears of corn plants of the Kculli race showing the arrangement of the grains per row. Ear samples from treatment T1 (**A**); treatment T2 (**B**); treatment T3 (**C**); treatment T4 (**D**); treatment T5 (**E**); treatment T6 (**F**); and treatment T7 (**G**).

In other studies carried out with purple corn plants of the INIA-601 and PVM-581 improved varieties, managed under a chemical fertilization scheme, an ear length of 16.637 to 20.76 cm was reported [79,84]. On the other hand, the ear lengths observed in the plants of the treatments T5 and T3 were statistically similar, registering values of 16.52 and 16.3 cm. These data are consistent with the values reported for the PMV-581, Arequipeño and INIA-615 varieties, with ear lengths of 16.26, 15.76 and 15.16 cm, respectively [57]. Slightly shorter ear lengths were obtained in Arequipeño purple corn plants, managed under three different distances (75, 80 and 85 cm) between furrows, with ear lengths of 14.5, 14.54 and 14.91 cm [90], respectively, which are similar to the value of 14.56 cm recorded in treatment T4. Finally, in treatment T1, the shortest ear length was recorded, with a value of 8.43 cm (Figure 5).

#### 3.4.5. Ear Diameter

For the ear diameter measurement, significant differences were observed between the treatments (*p* ≤ 0.05). The best results with values between 3.79, 3.7 and 3.62 cm were registered in the treatments T7, T5 and T6, respectively. Similar results were reported for the other varieties of purple corn grown in Peru, with an ear diameter between 3.38 and 3.78 cm [92]. The interaction between the corn plants and bioinoculants results in significant increases in the diameter of the ears, from 4.04 to 5.34 cm [61], which agrees with the ear diameters obtained in treatments T7 and T6. Smaller ear diameters between 3.52, 3.46 and 3.38 cm were observed in treatments T2, T4 and T3, respectively (Table 5). In this sense, under a scheme of chemical fertilization based on potassium and phosphorus, in the corn plants of the Canteño variety, values between 4.67 and 5.06 cm of ear diameter were reported [62]. Higher values of between 5.76 and 6.25 cm ear diameter were reported for purple maize plants of the Kculli race, where the largest diameters corresponded to plants with the largest ear size of 21.11 cm [73].

The difference in ear diameter observed between the ears of the treatments evaluated in this study and the reports consulted in the literature may be due to the fact that the ears of the corn plants in this research were long and thin, conical-cylindrical in shape, with few rows arranged slightly spirally or straight (Figure 5). In this sense, the improved purple corn varieties compensate for the loss of ear length with the increase in the diameter of the ear, which increases the size of the grain, obtaining small ears with large grains [73]. In addition, the greater the distance between the plants per row means that the ear diameter will increase, compared to smaller distances between plants, where the capture and interception of light and the photosynthetic rate is reduced [90]. In this regard, the distance of 25 cm selected between plants can explain the results recorded in this research, due to the less space available per plant, which resulted in a low assimilation of photosynthates in the ears and the reduction in their observed diameter. Finally, the smallest ear diameter was found in the treatment T1 plants, with a value of 2.9 cm, where nutrient deficiency resulted in small ears with few grains and rows (Table 5).

#### 3.4.6. Number of Grain Rows

In relation to this variable, the best results were observed in treatments T6, T5 and T4, with a total of 9.29, 9.09 and 9.01 grains per ear row, respectively. Other authors report superior values of between 10 to 12 rows for plants of improved purple corn and two populations of Kculli race, under an adequate fertilization scheme [73,79,88]. On the other hand, in the treatment T7, 8.99 rows of grains were registered, a value that is slightly lower than the values reported for the treatments that included bioinoculants and/or organic matter in their formulation. Lower values of 8.39 and 8.12 grain rows were recorded in treatments T2 and T3, respectively, fertilized with chemical fertilizer and/or bioinoculants plus chemical fertilizer. In this regard, for the chemically fertilized INIA-601 and INIA-606 varieties, a number of between 9.77 to 14 rows per ear was reported [75,84]. Although the number of grain rows recorded in treatments T7, T2 and T3 was slightly lower than that obtained in treatments T6, T5 and T4, the length of the ears, grain weight and grain size were higher in the treatments than in the formulation that included chemical fertilizer. Finally, the lowest number of rows of grains per row was found in treatment T1 (Table 5).

#### 3.4.7. Number of Grains Per Row

Regarding the number of grains per row, the best results were found in treatments T7 and T6, with a total of 20.37 and 19.82 grains per row, respectively. In this sense, for the maize plants of the Kculli race grown in Cusco, Peru, between 18.36 and 20.43 grains per row were reported [73]. Slightly higher numbers of 21.42 grains per row were reported for the starchy maize plants of the INIA-606 variety [75]. These data are consistent with the values reported in this work in treatments T7 and T6 for the same race of maize. On the other hand, for the INIA-601 variety of purple corn, higher values of between 26.15 and 28.67 grains per row were reported, corresponding to the plants cultivated under a chemical fertilization scheme, in Monsefu Lambayeque, Peru [84], where the main effect on the number of grains per row was due to the genetic material used. The treatments that received only one type of fertilizer in their formulation (Table 1) registered between 16.16 and 15.47 grains per row. Finally, the lowest results, with values of 13.74 and 9.17 grains per row, were recorded in treatments T3 and T1 (Table 5), respectively.

#### 3.4.8. One Hundred Grain Weight

Regarding the weight of 100 grains, the best results were obtained in treatments T7, T2 and T3 with weights of 45.6, 45.2 and 45 g, respectively. In this sense, for maize plants of the Kculli race, a weight of 100 grains of between 46.52 and 56.4 g was reported [73]. Higher values from 49.58 to 56.9 g for the weight of 100 grains were reported for the other varieties of purple corn that came from Peru [79,88,92]. On the other hand, in treatments T6, T4 and T5, weights of 100 grains of between 44 and 44.4 g were recorded (Table 5). In this regard, when corn plants were inoculated with bioinoculants, an increase of 14% in the mass of the thousand grains, evaluated in relation to the control, was observed [56,61], a result that is consistent with the results obtained in this study for treatments T6 and T4. Slightly lower values, between 37.9 and 47.1 g, were reported for various purple maize germplasm from Peru and Ecuador, respectively [19]. Finally, the lowest weight of 100 grains with a value of 23.8 g was recorded in treatment T1. Similar weights of 100 grains of 28 g have been reported in corn grains of the elotes conicos race with a dark blue grain color, grown in the state of Veracruz, Mexico [102]. Weights of 100 grains less than 19.6 g were reported for the purple maize plants from Costa Rica [19].

#### 3.4.9. Grain Length and Width

Long, medium-wide grains, often moderately imbricated, sometimes round, not indented, with white endosperm and a floury texture and rarely purple aleurone characterize this race of maize; pericarp and rachis colors are usually cherry-purple [74]. When analyzing the variables of grain length and width, it was observed that there were significant differences between the treatments evaluated (Table 6).

The best grain length and width were recorded in treatments T7 and T2, with values of 1.1 and 1.07 cm for grain length and 1.71 and 1.35 cm for grain width, respectively. For the corn of the Kculli race, grown in Peru, values for grain length of between 1.39 to 1.74 cm and 0.99 to 1.11 cm for grain width were recorded [73]. On the other hand, some races of corn from Mexico reported length values of 1.52, 1.48 and 1.16 cm and grain width of 0.86, 1.37 and 0.93 cm, respectively [103]. It is worth mentioning that these races have black, blue and/or purple grain, commonly used for the extraction of anthocyanins for culinary purposes. These data are similar to the values reported for treatments T7 and T2 in this work. On the other hand, for treatments T3, T4, T5 and T6, the grain length varied from 0.95 to 1.05 cm and the grain width from 1.13 to 1.29 cm. Other authors report a grain length of 0.62 cm and a grain width of 1.5 cm in maize plants of the conical corn race with a blue grain color, grown in Mexico [102]. Finally, the smallest grain length and width was recorded in treatment T1 (Table 6).

### 3.5. Proximal Chemical Analysis of Native Corn Starches

The starches of the corn samples corresponding to the seven treatments presented humidity percentages between 6.80 and 10.24%, results that are similar to the reports for the blue and hybrid corn starches with values of 6.5 and 8.5%, respectively [45]. The moisture content of the starches isolated in the different corn fertilization schemes analyzed in this study is within the generally accepted moisture range for dry products with a desirable shelf life, and is lower than the permitted value (<13%), based on NMX-F-382-1986 [104]. The observed moisture content was attributed to the high water/moisture retention capacity of corn starch in its cell wall. However, compared to the reports of other authors, for ashes of 0.1%, fat from 0.2 to 0.5%, and protein from 1.1 to 2.0% [105,106] the analyzed samples of purple corn grains showed a higher ash content, as well as a fat content within the average and a lower protein content (Table 7). The low ash, fat and protein contents in the chemical composition of corn starches, reported under the different fertilization schemes, indicate their high purity. In the literature, fertilization with N is reported as the main factor that determines the final concentration of grain protein [12,65,76]. In this sense, across all the treatments, a significant effect was found for treatments T7 and T3, which included chemical fertilizer, bioinoculants and/or organic matter within their formulation (Table 1). Likewise, other authors have reported that a low fat and protein content in a sample indicates the high purity of isolated starches [107]. The lipids and proteins in the starch granules can increase their functionality; protein in starch granules is associated with grain hardness, while lipids can significantly reduce the swelling capacity of starch paste. On the other hand, the presence of minerals in starch has been related to an increase in pasta clarity and viscosity. In addition, the low moisture content reduces the risk of microbial growth during storage [108,109]. However, the composition of corn can be modified by plant breeding methods to be used as a food source and in the wet milling and ethanol industries [110,111,112].

### 3.6. Fourier Transform Infrared Spectroscopy

**Figure 6:**
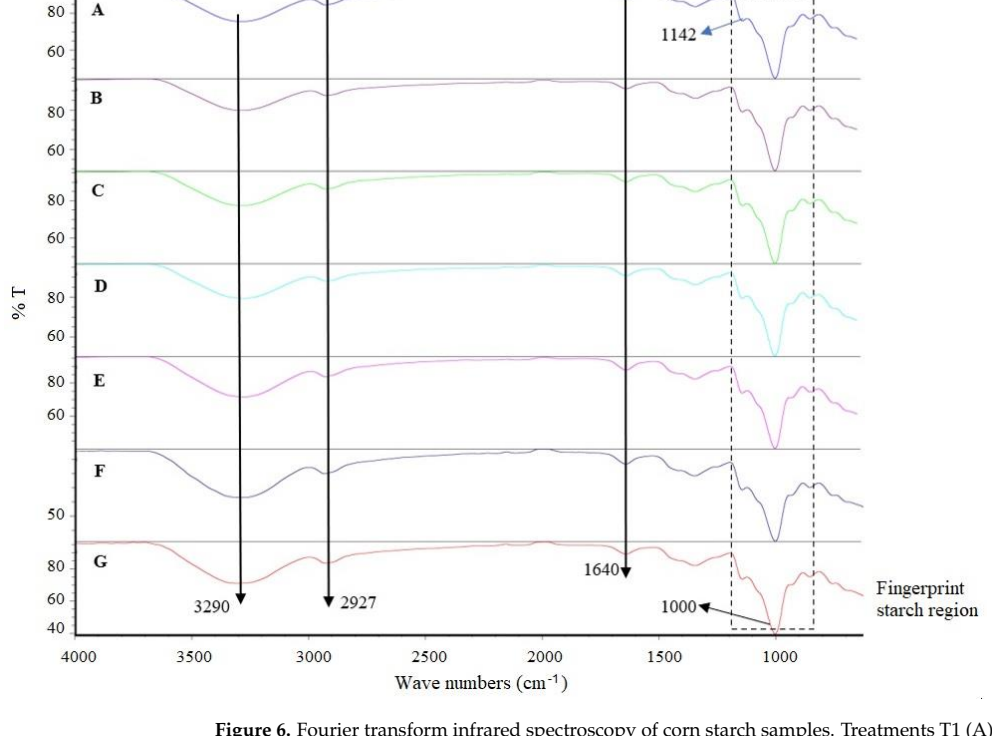
Fourier transform infrared spectroscopy of corn starch samples. Treatments T1 (A); T2 (B); T3 (C); T4 (D); T5 (E); T6 (F); and T7 (G).

A Fourier transform infrared (FTIR) spectra analysis of the native purple corn starches provides information on the arrangement of the functional organic groups and their interactions in the molecule. Since the infrared (IR) beam only penetrates the first few micrometers (approximately 2 µm) into the granule, it implies that the IR spectra are representative of the exterior of the starch granules [113]. Figure 6 represents the FTIR spectra of treatments T1–T7, where the starch fingerprint region (1250 to 500 cm^−1^) of the FTIR spectra was similar for the starches tested. 

The most intense peaks corresponded to carbohydrate vibrations, showing no apparent structural differences in the low rank molecular orders [114]. The peak observed at 1640 cm^−1^ corresponded to the strongly bound water molecules in the starches [115]. The region between 1200 and 800 cm^−1^ is characteristic of the C–O and C–C stretching and C–O–C strain modes associated with the glycosidic bond [116]. The spectra of the starch samples showed a peak at 3290 cm^−1^, which corresponds to the symmetric and asymmetric stretching of the O–H bonds [114]. On the other hand, the peak at 2927 cm^−1^ was attributed to the stretching of the C–H bond [117]. Finally, the peaks observed between 937 and 1156 cm^−1^ were attributed to stretching of the C–O bond [118]. The different fertilization schemes evaluated did not modify the molecular arrangement on the granule surface of the native maize starches.

### 3.7. Tristimulus Color

The luminosity factor (L*) was determined to measure the whiteness of the native starches of the purple corn grain samples of the treatments T1-T7 (Table 1), since this value (L*) is the best parameter to characterize the color of the starches and is a direct measure of their whiteness. The starches isolated from the purple corn grains showed a white color and values between 90.76 and 96.60. The starch of the purple corn grains corresponding to treatment T7 presented the highest whiteness, unlike the other starches obtained from the other treatments evaluated (Table 8), which is probably as a consequence of the residual anthocyanins of those samples [119]. The isolated starches, isolated from maize under different fertilization schemes, presented values similar to those of sorghum, rice and corn that showed L* values of 90.8 to 95.7 [120,121,122], but much lower than those reported for the hybrid, white and blue corn starches, with values of 93.6 to 98.4, respectively [45]. The finished color of the starch constitutes an important attribute of its quality, as the starches of white color have lower levels of protein and pigments. This explains the low protein content recorded in the grain starches of all the treatments evaluated in this research (Table 7). Likewise, the whiteness is an important physical parameter of the starches, since it determines their industrial applications in the pharmaceutical, food and agricultural sectors, among others [123], from which the starches isolated from the different fertilization schemes meet the characteristics for their application in the food industry.

## 4. Conclusions

It is evident that the supply of nutrients provided to the purple corn plants of the Kculli race, in each evaluated fertilization scheme, produced a significant yield increase of between 3.03 to 6.19 t ha^−1^, with respect to the yield obtained with the control of 1.02 t ha^−1^, which in percentage implies an increase from 197.05 to 506.86%. The treatment T7 presented the best yield (6.19 ± 0.07 t ha^−1^), surpassing all the other treatments evaluated. It was also the treatment that registered the highest grain protein content and quality of the evaluated starch. Therefore, this study provides alternatives and knowledge for the production of corn in a sustainable way for producers, at the local and national level. The rational use of renewable resources and the minimum use of materials and/or processes whose production and development entails the consumption of non-renewable energy sources, such as the use of chemical fertilizers, must be drastically reduced. The need to continue conducting studies aimed at identifying fungal-type biofertilizers with bioregulatory characteristics applied to agricultural crops must also be highlighted.

On the other hand, due to the health benefits [80,124] and nutrition [125] that the consumption of purple corn of the Kculli race entails for the human being, as well as the anti-inflammatory effect, tissue regeneration and collagen formation [126], the sowing of purple corn of the Kculli race, or its improved varieties, represents a profitable alternative for producers in the Apan region and in Mexico. This is because the worldwide trend is to consume natural products with nutritional properties that, in turn, have a benefit for the health of the consumer [127]. Additionally, biofertilizers are more profitable and useful products, since their cost is only 10% compared to the cost of a chemical fertilizer [128]. In addition, it constitutes an option to link the small producer with the national and international market, since with the sale of grain, rachis and bracts with a high content of anthocyanins, they would be able to increase their income.

Derived from the results obtained in this study, treatment T7, based on bioinoculants, organic matter and inorganic fertilization, could be a feasible alternative to implement among local producers because it is friendly to the environment, simple to apply and economical, since it reduces the use of agrochemical products and the high production costs involved. In addition, the reduction in the excessive use of chemical inputs, such as pesticides and synthetic fertilizers, will considerably decrease the loss of soil fertility, contamination of groundwater, air and soil, as well as damage to human health.

## Figures and Tables

**Table 1:** Different fertilization schemes used for the treatments evaluated in the growth and yield experiments.

Treatments	Key
Control	T1
Chemical fertilizer ^1^	T2
Chemical fertilizer + Bioinoculants	T3
Bioinoculants ^2^	T4
Organic fertilizer	T5
Organic fertilizer + Bioinoculants	T6
Chemical fertilizer + Bioinoculants + Organic fertilizer	T7

^1^ Adjusted rate based on fertilization levels required for one hectare for high N levels (210 kg ha^−1^); P_2_O_5_ (84 kg ha^−1^) and K_2_O (175 kg ha^−1^), without neglecting the annual addition of MgO (10 kg ha^−1^), S (37 kg ha^−1^), Zn (2.5 kg ha^−1^) and B (2 kg ha^−1^); ^2^ quantities adjusted according to the dose (400 g of product with CFU) necessary to apply in one hectare.

**Table 2:** Data obtained for the growth variables evaluated in the different fertilization schemes studied.

Key	Plant Height at Stage VT ^1^ (cm) ± SE ^2^	Stem Diameter (cm) ± SE
T1	185.63 ± 0.60 a	1.30 ± 0.42 a
T2	253.30 ± 0.07 b	2.02 ± 0.27 b
T3	263.33 ± 0.05 c	1.94 ± 0.33 ab
T4	283.46 ± 0.08 d	1.89 ± 0.42 ab
T5	262.70 ± 0.06 c	1.87 ± 0.30 ab
T6	275.43 ± 0.04 d	1.83 ± 0.30 ab
T7	315.34 ± 0.03 e	2.06 ± 0.21 b

^1^ Stage of vegetative development during which the maize plant has reached its final height and has fully exposed the panicle, prior to the start of pollen shedding in the R0 reproductive stage; ^2^ standard error, where the means in a column with the same literal are statistically similar (*p* ≤ 0.05, ANOVA and Tukey’s test).

**Table 3:** Values of the agronomic variables evaluated during the stage of reproductive development in purple corn plants, grown under different fertilization schemes.

	Stages R0 and R1 (Beginning of Pollen Shedding and Emergence of Silks on Ears)			Stage R6 (Physiological Maturity)
Key	MF ^1^ ± SE ^5^	FF ^2^ ± SE	FA ^3^ ± SE	Time to Harvest ^4^ (Days) ± SE
T1	118.67 ± 0.9 d	128.67 ± 1.0 d	10.00 ± 0.00 e	180.67 ± 1.53 e
T2	113.67 ± 0.5 c	122.00 ± 0.80 c	8.33 ± 0.50 d	169.00 ± 1.00 d
T3	100.67 ± 0.3 a	106.33 ± 0.40 a	5.67 ± 0.40 b	149.33 ± 1.53 a
T4	115.33 ± 0.2 c	123.67 ± 0.30 c	8.33 ± 0.50 d	166.67 ± 0.58 c
T5	111.00 ± 0.6 b	118.33 ± 0.30 b	7.33 ± 0.30 c	166.33 ± 1.15 c
T6	105.33 ± 0.4 b	112.67 ± 0.50 b	7.33 ± 0.30 c	154.67 ± 1.05 b
T7	98.67 ± 0.1 a	103.33 ± 0.20 a	4.67 ± 0.10 a	147.67 ± 0.23 a

^1^ Days to 50% male flowering; ^2^ days to 50% female flowering; ^3^ intervals between emission of pistils and anthesis; ^4^ time elapsed from germination and seedling emergence to physiological maturity during the reproductive development stage R6, with an average grain moisture of 30–35%; ^5^ standard error, where the differences in the letters within the same column indicate that they are significant at a level of *p* ≤ 0.05.

**Table 4:** Data derived from the yield variables evaluated in the fertilization schemes studied.

Key	Total Number of Plants ± SE ^1^	Number of Ears ± SE	Number of Ears per Plant	Ear Weight (g) ± SE	Grain Yield (t ha^−1^) ± SE
T1	81.00 ± 1.00 a	57.00 ± 1.00 a	0.70	25.15 ± 0.02 a	1.02 ± 0.01 a
T2	96.00 ± 0.00 b	111.60 ± 0.50 b	1.16	85.18 ± 0.08 b	4.35 ± 0.10 b
T3	96.00 ± 0.00 b	112.60 ± 0.50 b	1.17	71.63 ± 0.06 c	3.88 ± 0.07 c
T4	96.00 ± 0.00 b	92.00 ± 1.00 c	0.96	61.44 ± 0.04 d	3.03 ± 0.02 d
T5	91.30 ± 0.50 c	77.30 ± 0.50 d	0.85	72.91 ± 0.06 c	3.25 ± 0.06 c
T6	93.30 ± 0.50 c	84.60 ± 0.50 e	0.90	74.19 ± 0.05 c	3.46 ± 0.03 c
T7	96.00 ± 0.00 b	114.30 ± 0.50 b	1.19	96.62 ± 0.07 e	6.19 ± 0.07 e

^1^ Standard error, where means with the same letter do not differ significantly (*p* ≤ 0.05, ANOVA and Tukey’s test).

**Table 5:** Yield data for corn ears.

Key	Ear Diameter (cm) ± SE ^1^	Ear Length (cm) ± SE	Number of Rows per Ear ± SE	Number of Grains per Row ± SE	Weight of 100 Grains ± SE
T1	2.90 ± 0.58 c	8.43 ± 0.78 d	7.68 ± 2.58 d	9.17 ± 0.61 e	23.80 ± 0.15 a
T2	3.52 ± 0.75 b	17.20 ± 0.59 b	8.39 ± 2.13 c	16.16 ± 0.54 c	45.20 ± 0.60 bc
T3	3.38 ± 0.81 b	16.30 ± 0.10 bc	8.12 ± 2.39 c	13.74 ± 0.93 d	45.00 ± 0.12 bc
T4	3.46 ± 0.56 b	14.56 ± 0.92 c	9.01 ± 1.46 a	15.47 ± 0.04 c	44.10 ± 0.08 b
T5	3.70 ± 0.62 a	16.52 ± 0.91 bc	9.09 ± 1.62 a	16.00 ± 0.51 c	44.00 ± 0.11 b
T6	3.62 ± 0.58 ab	17.25 ± 0.23 b	9.29 ± 1.61 a	19.82 ± 0.66 b	44.40 ± 0.12 b
T7	3.79 ± 0.56 a	18.95 ± 0.77 a	8.99 ± 1.48 b	20.37 ± 0.18 a	45.60 ± 0.05 bc

^1^ Standard error, where means with the same letter do not differ significantly (*p* ≤ 0.05, ANOVA and Tukey’s test).

**Table 6:** Data on the length and grain width of the ears of the Kculli race.

Key	Grain Length (cm) ± SE ^1^	Grain Width (cm) ± SE
T1	0.81 ± 0.02 a	0.54 ± 0.07 a
T2	1.07 ± 0.01 c	1.35 ± 0.04 b
T3	1.02 ± 0.01 bc	1.29 ± 0.01 b
T4	1.03 ± 0.01 bc	1.15 ± 0.01 c
T5	1.05 ± 0.04 bc	1.13 ± 0.01 c
T6	0.95 ± 0.07 b	1.14 ± 0.02 c
T7	1.10 ± 0.01 c	1.71 ± 0.03 d

^1^ Standard error, where means with the same letter do not differ significantly (*p* ≤ 0.05, ANOVA and Tukey’s test).

**Table 7:** Physicochemical properties of native corn starches.

	Moisture % ± SE ^1^	Ash % ± SE	Fat % ± SE	Protein % ± SE
T1	7.94 ± 0.06 c	0.35 ± 0.03 a	0.27 ± 0.05 a	0.37 ± 0.03 ab
T2	7.93 ± 0.27 c	0.43 ± 0.09 a	0.31 ± 0.11 a	0.39 ± 0.02 ab
T3	7.97 ± 0.12 c	0.46 ± 0.07 a	0.35 ± 0.07 a	0.35 ± 0.01 b
T4	10.24 ± 0.09 a	0.35 ± 0.04 a	0.36 ± 0.07 a	0.39 ± 0.00 ab
T5	8.96 ± 0.16 b	0.41 ± 0.05 a	0.37 ± 0.02 a	0.39 ± 0.02 ab
T6	6.92 ± 0.05 d	0.41 ± 0.04 a	0.34 ± 0.03 a	0.39 ± 0.00 ab
T7	6.80 ± 0.65 d	0.35 ± 0.03 a	0.36 ± 0.03 a	0.41 ± 0.00 a

^1^ Standard error, where means with the same letter do not differ significantly (*p* ≤ 0.05, ANOVA and Tukey’s test).

**Table 8:** Color analysis.

	L ± SE ^1^	a ± SE	b ± SE
T1	91.76 ± 0.45 bc	0.90 ± 0.02 a	2.40 ± 0.05 b
T2	90.76 ± 0.28 c	0.93 ± 0.02 a	2.75 ± 0.05 a
T3	92.69 ± 0.41 b	0.15 ± 0.02 e	2.42 ± 0.06 b
T4	91.98 ± 1.11 bc	0.15 ± 0.02 e	2.31 ± 0.14 bc
T5	92.44 ± 0.57 b	0.20 ± 0.02 d	2.35 ± 0.07 b
T6	90.77 ± 1.08 c	0.25 ± 0.01 c	2.15 ± 0.14 c
T7	96.60 ± 0.70 a	0.41 ± 0.02 b	1.60 ± 0.06 d

^1^ Standard error, where means with the same letter do not differ significantly (*p* ≤ 0.05, ANOVA and Tukey’s test).

## Data Availability

Not applicable.
